# A Novel Recombinant MAGE-B10-HSP110 Fusion Protein Enhances Innate and Adaptive Immune Responses in Mice: A Potential Vaccine Candidate for Canine Mammary Tumors

**DOI:** 10.3390/ani16091374

**Published:** 2026-04-29

**Authors:** Anucha Muenthaisong, Kanokwan Sangkakam, Pongpisid Koonyosying, Thanya Varinrak, Nattawooti Sthitmatee

**Affiliations:** 1 Laboratory of Veterinary Vaccine and Biological Products, Faculty of Veterinary Medicine, Chiang Mai University, Chiang Mai 50100, Thailand; anucha.m@cmu.ac.th (A.M.); kanokwansangkakam@gmail.com (K.S.); pongpisid_koo@nation.ac.th (P.K.); thanya.var@cmu.ac.th (T.V.); 2 Office of Research Administration, Chiang Mai University, Chiang Mai 50200, Thailand; 3 Faculty of Medicine, Nation University, Chiang Mai 50210, Thailand; 4 Veterinary Diagnostic and Technology Transfer Center, Faculty of Veterinary Medicine, Chiang Mai University, Chiang Mai 50100, Thailand

**Keywords:** canine mammary tumor, heat shock protein 110, melanoma-associated antigen B10, recombinant fusion canine MAGE-B10 and HSP110 protein

## Abstract

Melanoma-associated antigen (MAGE) is a promising immunotherapeutic target for cancer vaccines. Heat shock protein 110 (HSP110), expressed in various tumors, including canine mammary tumors, serves as a molecular marker. This study aimed to develop a recombinant fusion protein by linking HSP110 with MAGE-B10 to target MAGE-B10-expressed tumors and assess immune response efficacy. The recombinant MAGE-B10-HSP110 (rMAGE-B10-HSP110) fusion protein was constructed, and separate recombinant MAGE-B10 (rMAGE-B10) and recombinant HSP110 (rHSP110) proteins were also prepared for comparison. Our study on mice is distributed across five treatment groups: the rMAGE-B10-HSP110 fusion protein, rMAGE-B10, rHSP110, a protein mixture, and a PBS control. Antibody responses specific to canine MAGE-B10 were measured using ELISA, while splenocyte activation, proliferation, and cytokine production were analyzed using flow cytometry. The results showed significantly higher antibody responses in mice immunized with the rMAGE-B10-HSP110 fusion protein compared to those receiving PBS or rHSP110 on days 7, 14, and 21. The proportion of CD3+ and CD4+ lymphocytes were significantly higher in these mice (*p* < 0.05). rMAGE-B10-HSP110 fusion protein immunization also resulted in increased CD69+ lymphocytes and higher IFN-γ levels in stimulated lymphocytes (*p* < 0.05). In conclusion, the rMAGE-B10-HSP110 fusion protein effectively stimulates both innate and adaptive immune responses. Further in vivo investigation is recommended.

## Simple Summary

Melanoma-associated antigen (MAGE) is a promising immunotherapeutic target for cancer vaccines. Heat shock protein 110 (HSP110), expressed in various tumors, including canine mammary tumors, serves as a molecular marker. This study tested a candidate canine mammary cancer vaccine by combining a tumor target (MAGE) with a biological booster (HSP110) to create a fusion protein. By physically linking these two components, researchers aimed to better train the immune system to recognize and attack cancer cells. When tested in mice, this fusion protein performed significantly better than using the proteins separately or as a simple mixture. It triggered a more powerful double-threat response: The mice produced more specialized antibodies to hunt down the tumor and generated a higher number of active T cells to fight the disease. Ultimately, our study concludes that this fused protein is highly effective for jumpstarting a full-scale immune defense, paving the way for future cancer treatment trials.

## 1. Introduction

Canine mammary tumors (CMTs) are common in tissue samples obtained from intact female dogs, with higher incidence rates compared to other tumor types in dogs [1,2]. Previous studies have shown that the pathological characteristics of CMTs are similar to those of human breast cancer [3,4,5]. Traditional treatments such as surgery and chemotherapy often cause significant changes in a patient’s condition, especially in advanced tumors, resulting in severe pain, stress, and a poor quality of life. In malignant CMTs, residual tumor cells may remain after surgery and metastasize to distant organs such as the lymph nodes or lungs, contributing to patient mortality. Recurrence rates after surgery have been reported to reach 90% in poorly differentiated malignant CMTs [6]. Additionally, the effectiveness of chemotherapeutic agents has been minimally reported, and many side effects persist [7,8,9,10]. These findings highlight the need for alternative treatment options. Immunotherapy, which stimulates lymphocyte function, particularly cytotoxic T lymphocytes and natural killer cells, offers a promising approach for cancer treatment [11].

Melanoma-associated antigen (MAGE) has been identified as an ideal target for immunotherapeutic vaccines for human cancers. MAGE-1 is recognized by cytotoxic T lymphocytes via major histocompatibility complex class I (MHC class I) and contributes to immune system stimulation [12,13,14,15,16]. MAGE-1 expression has been reported in brain, lung, pancreatic, ovarian, and breast cancers [17,18,19]. In human breast cancer, MAGE-1 expression is linked to poor prognosis [20,21]. The MAGE-B family, including MAGE-B10, is not expressed in normal tissues except for certain germ cells. Previous studies have shown that MAGE-B DNA vaccines have the potential to prevent metastasis in breast cancer mouse models [22]. However, the effectiveness of these vaccines in primary tumor regression remains limited, suggesting that fusion proteins could improve vaccine efficacy.

Heat shock protein 110 (HSP110) is a molecular marker protein expressed in various human cancers, including breast cancer. Recent studies have shown the high expression of HSP110 in CMTs [23]. This protein binds to antigen-presenting cells via scavenger receptors, enhancing the immune response by activating naïve T cells [24]. This study hypothesizes that fusing MAGE-1 with HSP110 could enhance the efficacy of the protein against MAGE-1-expressing tumors, including CMTs. Our preliminary studies on MAGE gene expression revealed that MAGE-B1, -B4, -B5, and -B10 mRNA are expressed in CMTs [25]. Western blot and immunohistochemistry results confirmed the presence of a 51 kDa nuclear protein of canine MAGE antigen in CMTs, cross-reacting with the mouse anti-human MAGE-1 monoclonal antibody [26]. According to the level of mRNA expression, the canine MAGE-B10 gene was chosen as the candidate vaccine for this study.

Based on mRNA expression levels, the canine MAGE-B10 gene was selected as the candidate protein for this study. This study aims to develop a fusion protein prototype by linking HSP110 with MAGE-B10 to assess whether this fusion protein can enhance potency against MAGE-B10-expressing tumors and evaluate the immune response specificity to canine MAGE-B10 proteins in vitro.

## 2. Materials and Methods

### 2.1. Mice

Seventy female BALB/c mice, aged 6–8 weeks (Nomura Siam International Co., Ltd., Bangkok, Thailand), were maintained under specific pathogen-free conditions and provided laboratory-grade food and water ad libitum. This study was conducted in accordance with the guidelines of the Institute of Animals for Scientific Purposes Development (IAD), National Research Council of Thailand (NRCT); moreover, it was approved by the Animal Care and Use Committee (FVM-ACUC), Faculty of Veterinary Medicine, Chiang Mai University. The Chiang Mai University Institutional Biosafety Committee (CMU-IBC) reviewed and approved the project, confirming its compliance with biosafety guidelines for modern biotechnology, pathogens, and toxic agents.

### 2.2. Construction of the Protein Expression Plasmid and Protein Expression

**Figure 1:**
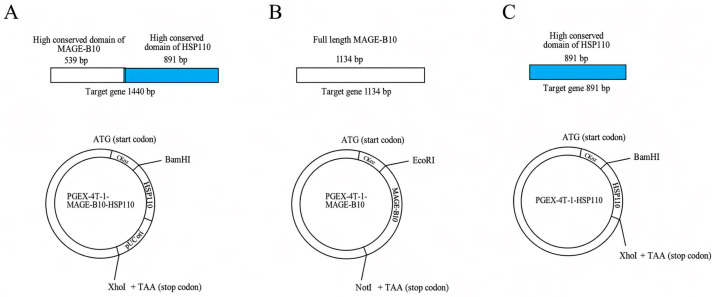
Schematic representation of the rMAGE-B10 and HSP110 fusion protein vaccine and comparative plasmid maps. (**A**) pGEX-4T-1-MAGE-B10-HSP110 fusion construct, (**B**) pGEX-4T-1-MAGE-B10 control plasmid, and (**C**) pGEX-4T-1-HSP110 control plasmid.

The conserved MAGE-B10 fragment used in the fusion construct was selected for antigenic conservation; however, because this fragment could not be expressed successfully as a standalone recombinant protein in the pGEX-4T-1/BL21 system, full-length MAGE-B10 was used as the comparative recombinant MAGE-B10 protein. The recombinant fusion MAGE-B10&HSP110 plasmid (pGEX-4T-1-MAGE-B10-HSP110) and the comparative plasmids, canine MAGE-B10 (pGEX-4T-1-MAGE-B10) and canine HSP110 (pGEX-4T-1-HSP110), were developed in our laboratory. Briefly, the canine MAGE-B10 gene was amplified via polymerase chain reaction (PCR) from the total RNA extracted from MAGE-B10-expressing canine mammary tumor (CMT) tissues, which were converted to cDNA. Primers cMAGE-B10-F and cMAGE-B10-R were used to screen MAGE-B10-expressing CMT samples, while primers pGEX-mage-F and pGEX-mage-R were used to amplify the target MAGE-B10 gene for insertion into the pGEX-4T-1 vector (Table 1). The amplified full-length MAGE-B10 product was digested with *Eco*RI (New England BioLabs, Ipswich, MA, USA) and *Not*I (New England BioLabs) before being cloned into pGEX-4T-1 (GenScript Biotech Corp, Piscataway, NJ, USA) at the C-terminal end of the GST tag sequence (Figure 1).

The recombinant fusion MAGE-B10 and HSP110 protein (rMAGE-B10-HSP110 fusion protein) was prepared using the highly conserved MAGE-B10 gene domain (GenBank accession number NM_001003116.1), fused with the highly conserved L and H domains of the HSP110 gene (GenBank accession number XP_013962905.1). *Bam*HI (New England BioLabs) and *Xho*I (New England BioLabs) restriction sites were added to the 5′ and 3′ ends of the selected MAGE-B10 and HSP110 genes (1st BASE™, Axil Scientific, Jurong West, Singapore). The target fusion MAGE-B10&HSP110 gene was digested with *Bam*HI and *Xho*I before being cloned into the pGEX-4T-1 vector at the C-terminal end of the GST tag sequence (Figure 1). Similarly, the L and H domains of the HSP110 gene were separately constructed by adding *Bam*HI and *Xho*I to the 5′ and 3′ ends, respectively. The target HSP110 gene was also digested with *Bam*HI and *Xho*I before being cloned into the pGEX-4T-1 vector at the C-terminal end of the GST tag sequence (Figure 1).

The pGEX-4T-1-MAGE-B10, pGEX-4T-1-MAGE-B10-HSP110, and pGEX-4T-1-HSP110 plasmids were then isolated and transformed into BL21 (DE3) competent *E. coli* (Invitrogen™, Thermo Fisher Scientific, Waltham, MA, USA) using heat shock transformation [27]. The peptide sequences of the proteins were predicted using the Phyre2 web portal, available at http://www.sbg.bio.ic.ac.uk/phyre2 (accessed on 23 November 2024) [28].

The transformed pGEX-4T-1-MAGE-B10-HSP110, pGEX-4T-1-MAGE-B10, and pGEX-4T-1-HSP110 plasmids were cultured in S.O.C. media and incubated at 37 °C with shaking at 200 rpm for 1 h. The cells were then plated on Luria–Bertani (LB) agar containing 100 μg/mL ampicillin for selection. Single white colonies were selected and cultured in LB broth with 100 μg/mL ampicillin at 37 °C for 12–16 h with shaking at 200 rpm until the optical density (OD) at 600 nm reached 0.5–0.6. The presence of the target gene in the plasmids was confirmed via double enzyme digestion, PCR, and DNA sequencing.

Each plasmid-containing *E. coli* strain was cultured in LB broth, and protein expression was induced with 0.5 mM isopropyl β-D-1-thiogalactopyranoside (IPTG; Invitrogen™). The culture was then incubated at 37 °C with continuous shaking at 125 rpm for 24 h [29]. Cell pellets were collected by centrifugation at 4000× *g* for 20 min. The rMAGE-B10-HSP110 fusion protein was harvested and confirmed via SDS-PAGE and Western blot analysis using an anti-GST monoclonal antibody (Invitrogen™). The target sizes of the rMAGE-B10-HSP110 fusion protein, recombinant HSP110 (rHSP110), and recombinant MAGE-B10 (rMAGE-B10) proteins with GST tags were estimated using Oligo 7 primer analysis software (Molecular Biology Insights, Inc., Colorado Springs, CO, USA), and they were approximately 95 kDa, 60 kDa, and 78 kDa, respectively.

The pGEX-4T-1-MAGE-B10-HSP110, pGEX-4T-1-MAGE-B10, and pGEX-4T-1-HSP110 plasmids—after size confirmation—were extracted and stored at −80 °C for subsequent steps. Similarly, the cells were supplemented with 30% glycerol and stored at −80 °C for future large-scale expression.

### 2.3. Protein Purification

Protein purification and the removal of the glutathione S-transferase (GST) tag were adapted from the Harper and Speicher method [30]. Briefly, large-scale protein expression in pGEX-4T competent *E. coli* was induced with IPTG solution. The cell pellets were harvested and washed with PBS. Protein extraction was performed using NP-40 lysis buffer supplemented with a protease inhibitor cocktail (MilliporeSigma, St. Louis, MO, USA). The target protein was purified using the GST fusion protein purification kit (Genscript Biotech), in accordance with the manufacturer’s instructions. After purification, the GST tags were removed using the thrombin cleavage capture kit (MilliporeSigma), following the manufacturer’s recommendations. Briefly, each purified recombinant protein was digested with biotinylated thrombin protease and incubated for 16 h at 22 °C. Streptavidin agarose was then added to the reaction to facilitate thrombin removal. Excess GST tags were removed by re-passing the protein through the GST purification column. The GST-free proteins were collected. Excess water was removed using Ultra 15 mL filters for protein purification and concentration (Amicon^®^, MilliporeSigma). Protein concentrations were measured using a BCA assay kit (Invitrogen™). The recombinant proteins were stored at −20 °C until use.

### 2.4. Immunization of Mice

A total of 35 mice were subdivided into five subgroups of seven mice each. Vaccine formulations were prepared by mixing different recombinant proteins with the Montanide™ ISA 206 VG adjuvant (1:1 V/V, SEPPIC, Paris, France) to achieve the total volume per dose, as described in Table 2. The vaccines included the following: (1) rMAGE-B10-HSP110 fusion protein: a fusion recombinant MAGE-B10 and HSP110 protein; (2) rMAGE-B10: a recombinant MAGE-B10 protein; (3) rHSP110: a recombinant HSP110 protein; (4) a mixture of rMAGE-B10 and rHSP110: a mixture of recombinant MAGE-B10 and recombinant HSP110 protein; (5) PBS: phosphate-buffered saline (PBS). The vaccines were freshly prepared and stored at 4˚C until use. Seventy mice were equally divided into five groups, as described in Table 2. Each mouse was immunized intramuscularly, followed by a booster on day 7 after the initial immunization. Sera were collected from the submandibular vein on days 0, 7, 14, and 21. The mice were sacrificed two weeks after the booster immunization. Spleens were collected aseptically, kept in cold PBS, and promptly processed for single splenocyte isolation.

### 2.5. Antibody Evaluation Using ELISA

Since this study aimed to determine immune responses targeting MAGE-B10-expressed CMTs, indirect ELISA was used to determine antibodies against canine MAGE-B10 proteins in the sera. First, 96-well flat-bottom ELISA plates were coated with 50 µL of 2.5 µg/mL purified full-length recombinant canine MAGE-B10 (rMAGE-B10) protein derived from the pGEX-4T-1-MAGE-B10 construct containing the 1134 bp MAGE-B10 cDNA insert and incubated overnight at room temperature. Next, the plates were rinsed with PBS and incubated with a blocking buffer (5% non-fat dry milk powder and 0.2% Tween 20 in PBS) for 2 h at 37 °C. After blocking, the mouse serum diluted 1:50 in the blocking buffer was added to the plates, and the plates were incubated for 2 h at 37 °C. The plates were then rinsed with PBS and incubated with horseradish peroxidase-conjugated anti-mouse IgG (Invitrogen™) for 1 h at 37 °C. Following this step, the plates were extensively washed, and tetramethylbenzidine (TMB; Invitrogen™) substrate was added. The plates were incubated at room temperature for 20 min. Finally, the reactions were stopped with 2 M H_2_SO_4_, and the ELISA plates were read at 450 nm.

### 2.6. CD4, CD3, CD69, and IFN-γ Measurement Using Flow Cytometer

The spleens of mice were collected, homogenized, and processed into single splenocytes using a 70 µm cell strainer (Thermo Fisher Scientific). The splenocytes were washed once with PBS, and red blood cells were removed using an RBC lysis buffer solution (Merck Millipore, Burlington, MA, USA) following the manufacturer’s instructions. The splenocytes were then resuspended in culture media consisting of RPMI 1640 with L-glutamine (Gibco™, Thermo Fisher Scientific) supplemented with 10% heat-inactivated fetal bovine serum (FBS; Gibco™), 1% penicillin, 1% streptomycin, 1% L-glutamine, and 0.1% β-mercaptoethanol.

Cell viability was assessed under a light microscope using trypan blue and a hemocytometer [31]. The splenocytes were counted and cultured in a 24-well plate with 500 μL of culture media, seeding 8 × 10^5^ cells per well, at 37 °C in a 5% CO_2_ atmosphere for 24 h. After incubation, the media were replaced with 400 μL of fresh culture media. The splenocytes were re-stimulated with 1 µL of 10 µg/mL purified full-length recombinant canine MAGE-B10 (rMAGE-B10) protein derived from the pGEX-4T-1-MAGE-B10 construct containing the 1134 bp MAGE-B10 cDNA insert in 100 µL of medium for 24 h at 37 °C in a 5% CO_2_ atmosphere. Positive and negative controls included 4 µg/mL phytohaemagglutinin (PHA; Gibco™) in culture medium and serum-free medium, respectively. The splenocyte response was quantified by assessing the number of CD4, CD3, and CD69 cell surface markers and measuring IFN-γ production by Type 1 helper T (Th1) cells using fluorescence-activated cell sorting (FACS), a specialized flow cytometry technique. For FACS analysis, splenocytes were harvested and incubated with brefeldin A (BD Biosciences, Franklin Lakes, NJ, USA) for 4 h. Each test sample was treated with 20 μL of anti-FcγII/III receptor monoclonal antibodies (MAbs) (BD Biosciences) and incubated on ice for 20 min. Subsequently, the splenocytes were labeled with fluorescent-conjugated antibodies (eBioscience™, San Diego, CA, USA) specific for mice, including CD3 (allophycocyanin (APC); clone 17A2) at 0.5 μg/test, CD4 (PE-Texas Red (ECD); clone RM4-5) at 0.25 μg/test, and CD69 (PE-Cyanine 7 (PC7); clone FN50) at 0.25 μg/test. The labeled cells were incubated on ice with light protection for 60 min and washed twice with a flow cytometry staining buffer (cold PBS + 1% FBS). The cells were then resuspended in serum- and protein-free PBS and labeled with a viability dye (LIVE/DEAD™ Fixable Dead Cell Stain Kits; Thermo Fisher Scientific) on ice with light protection for 30 min. Intracellular staining was performed using fluorescent-conjugated antibodies (eBioscience™) specific for IFN-γ (R-phycoerythrin (PE); clone XMG1.2) at 0.25 μg/test. Sample acquisition (10,000 events per sample) was conducted at a flow rate of 10 μL/min using a CyAn™ ADP Flow Cytometer (Beckman Coulter, Inc., Brea, CA, USA). The expression of CD3, CD4, and CD69 on lymphocytes in the spleens of mice was analyzed, and intracellular IFN-γ production was compared.

### 2.7. Statistical Analysis

The sample size of mice was calculated using G*Power 3.1.9.2 software to perform statistical power analyses. The means and standard errors of the mean (SEM) of optical density (OD) values for antibody titers in mouse sera were compared between treatment groups using analysis of variance (ANOVA) with repeated measures, conducted with GraphPad Prism version 10.2.0 (GraphPad Software, Inc., San Diego, CA, USA).

The proportions of CD3+, CD4+, and CD69+ lymphocytes were reported as percentages with their SEM, analyzed descriptively, and compared across protein types using ANOVA. Quantitative cytokine levels were also compared across protein types and between treatment (canine MAGE-B10 protein stimulation) and control groups (no canine MAGE-B10 stimulation) using ANOVA. Outlier data were excluded from the analysis. Statistical significance was defined as *p* < 0.05.

## 3. Results

### 3.1. Canine MAGE-B10 Expression in CMT Tissues

**Figure 2:**
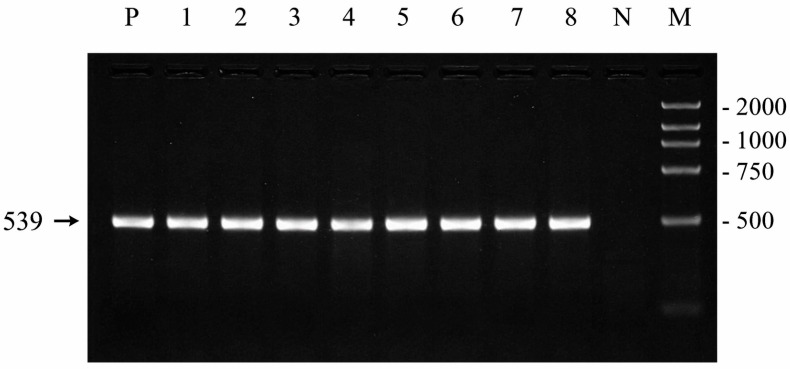
RT-PCR analysis of MAGE-B10 mRNA expression in canine mammary tumor tissues. Lane M: DNA marker; Lane P: positive control (testicular tissue); Lanes 1 and 5–7: simple tubulopapillary carcinoma; Lanes 2–4: complex carcinoma; Lane 8: mixed tumor carcinoma; Lane N: negative control (PBS). The arrow indicates the target MAGE-B10 amplification product at 539 bp.

The amplification results confirmed that all total cDNA samples used to construct the canine MAGE-B10-expressing plasmid (pGEX-4T-1-MAGE-B10) were derived from MAGE-B10-expressing CMT tissues (Figure 2).

### 3.2. pGEX-4T-1-MAGE-B10-HSP110 Plasmid and rMAGE-B10-HSP110 Fusion Protein

The sequencing results of the pGEX-4T-1-MAGE-B10-HSP110 plasmid confirmed that it contains the highly conserved domain of the canine *MAGE-B10* gene and the highly conserved L and H domains of the *HSP110* gene, inserted into the pGEX-4T-1 vector at the C-terminal end of GST.

**Figure 3:**
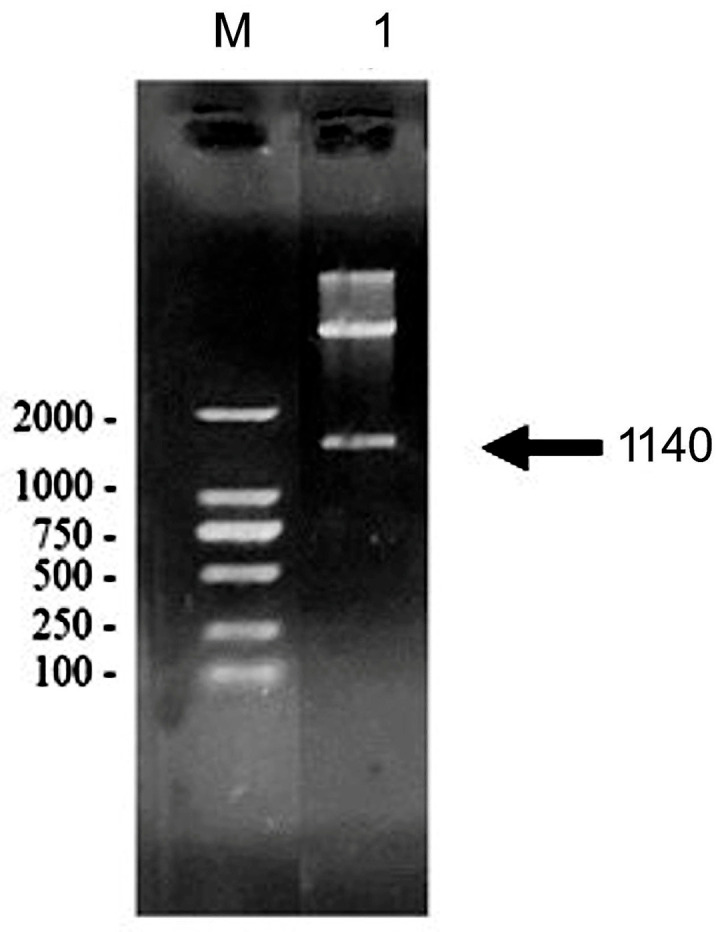
Confirmation of the pGEX-4T-1-MAGE-B10-HSP110 recombinant plasmid via restriction analysis. Agarose gel electrophoresis shows the result of a double digestion of the pGEX-4T-1-MAGE-B10-HSP110 construct using *Eco*RI and *Xba*I. Lane M: DNA marker; Lane 1: released MAGE-B10-HSP110 fusion insert. The arrow indicates the target fragment at approximately 1140 bp.

**Figure 4:**
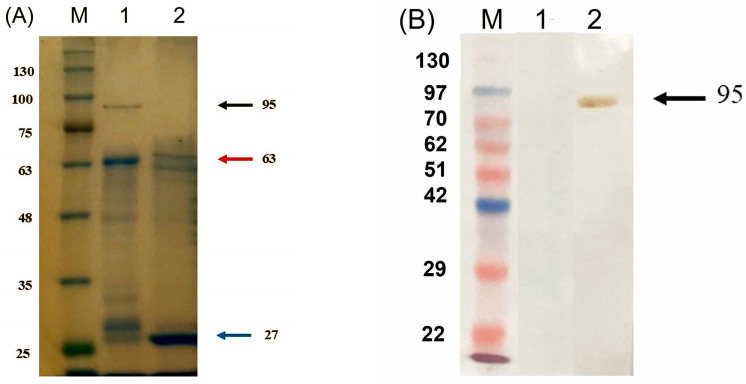
Expression and proteolytic cleavage analysis of the recombinant MAGE-B10-HSP110 fusion protein. (**A**) SDS-PAGE analysis of the proteolytic cleavage of the purified protein. Lane M: Molecular weight marker; Lane 1: purified GST-tagged MAGE-B10-HSP110 prior to cleavage; Lane 2: fusion protein following digestion with biotinylated thrombin. Arrows indicate the intact fusion protein (~95 kDa), the cleaved MAGE-B10-HSP110 (~63 kDa), and the released GST tag (~27 kDa). (**B**) Western blot analysis of fusion protein expression in *E. coli* BL21. Proteins were detected using an HRP-conjugated anti-GST monoclonal antibody (1:2000). Lane M: Molecular weight marker; Lane 1: non-induced total protein lysate (control); Lane 2: total protein lysate induced with 0.5 mM IPTG for 24 h. The arrow indicates the GST-tagged MAGE-B10-HSP110 fusion protein at the expected molecular weight (~95 kDa).

The electrophoresis results from double enzyme digestion confirmed the presence of the target MAGE-B10-HSP110 DNA fragment at approximately 1440 base pairs (Figure 3). SDS-PAGE analysis indicated that the sizes of the target rMAGE-B10-HSP110 fusion protein with and without the GST tag were approximately 95 kDa and 63 kDa, respectively (Figure 4A). Western blotting results showed the size of the target rMAGE-B10-HSP110 fusion protein with the GST tag (Figure 4B).

### 3.3. pGEX-4T-1-MAGE-B10 Plasmid and rMAGE-B10

The highly conserved domain of the *MAGE-B10* gene, identical to the fragment used in the previously described fusion protein, failed during the protein expression process using the pGEX-4T-1 and BL21 *E. coli* system. Consequently, the full-length canine *MAGE-B10* gene was selected and constructed in our laboratory using cDNA obtained from canine MAGE-B10-positive CMT tissues as the DNA template.

**Figure 5:**
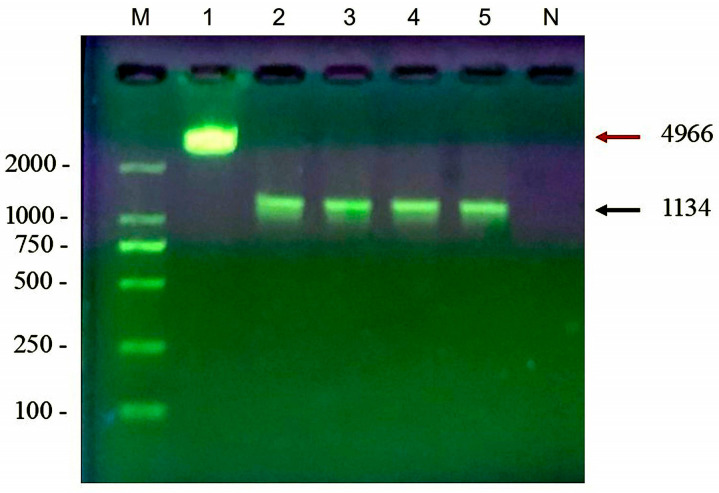
Verification of linearized pGEX-4T-1 vector and MAGE-B10 PCR products via restriction analysis. Lane M: DNA marker; Lane 1: pGEX-4T-1 vector linearized by *Eco*RI and *Not*I double digestion; Lanes 2–5: *Eco*RI/*Not*I double-digested MAGE-B10 PCR products; Lane N: negative control. The red arrow indicates the linearized pGEX-4T-1 vector at 4966 bp, while the arrow denotes the digested canine MAGE-B10 insert at 1134 bp.

The target MAGE-B10 DNA, flanked by *Eco*RI and *Not*I restriction sequences at the 5′ and 3′ ends, respectively, was amplified using the pGEX-mage-F and pGEX-mage-R primer pairs (Table 1). The amplified 1134 base pair DNA fragment was electrophoresed and purified (Figure 5). The pGEX-4T-1 vector was digested with *Eco*RI and *Not*I restriction enzymes to generate a linearized pGEX-4T-1 vector for gene insertion. Subsequently, the 4966 base pair linear pGEX-4T-1 vector was electrophoresed and purified.

**Figure 6:**
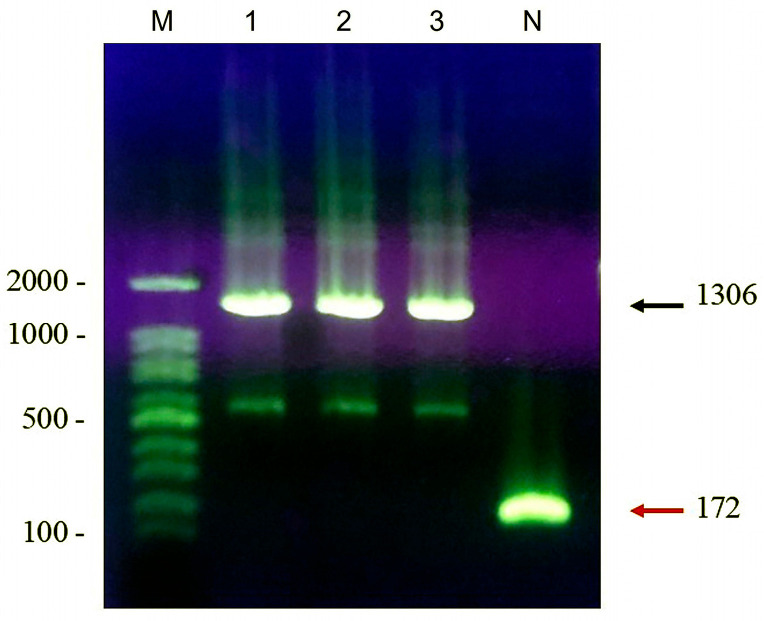
PCR verification of MAGE-B10 insertion into the pGEX-4T-1 vector. Lane M: DNA marker; Lanes 1–3: PCR products from pGEX-4T-1-MAGE-B10 recombinant plasmids isolated from *E. coli* BL21 colonies 1–3; Lane N: negative control (empty pGEX-4T-1 vector). The arrows indicate the recombinant amplicon at 1306 bp and the empty vector amplicon at 172 bp.

The ligation of the target gene and plasmid was performed, and the success of the ligation was confirmed via PCR amplification using pGEX universal primers. The target DNA of the pGEX-4T-1-MAGE-B10 plasmid was 1306 base pairs, while the target DNA of the free pGEX-4T-1 plasmid was 172 base pairs (Figure 6). The sequencing results confirmed that the full-length *MAGE-B10* gene was successfully inserted into the pGEX-4T-1 vector at the C-terminal end of GST. The SDS-PAGE results indicated that the size of the rMAGE-B10 with the GST tag was approximately 68 kDa (Supplementary Figure S1), while the final rMAGE-B10, after GST tag removal, was approximately 41 kDa. Additionally, Western blotting results confirmed that the rMAGE-B10 with the GST tag was approximately 68 kDa (Supplementary Figure S2).

### 3.4. Antibody Response to Protein Candidates

**Figure 7:**
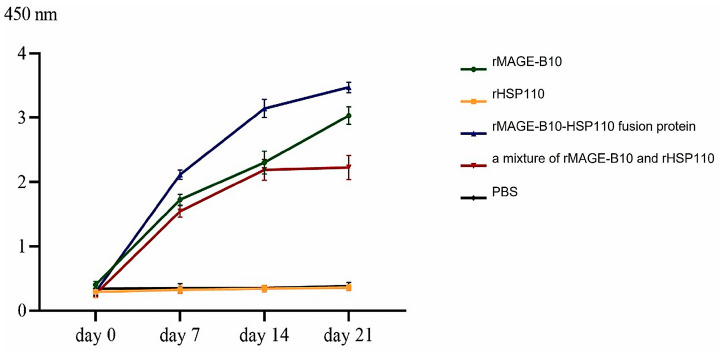
Longitudinal analysis of serum antibody responses to canine MAGE-B10 in immunized mice. Serum samples were collected on days 0, 7, 14, and 21 post-immunization to compare the immunogenicity of the candidate vaccines. Data are presented as [mean ± SD].

The present study revealed that the mean level of antibodies specific to canine MAGE-B10 in mice immunized with an rMAGE-B10-HSP110 fusion protein increased after the second immunization. Its levels tended to continue rising on days 7, 14, and 21. Similarly, the mean antibody levels in mice immunized with rMAGE-B10 and a mixture of rMAGE-B10 and rHSP110 increased in parallel (Figure 7). Comparing the groups at days 14 and 21 post-immunization, the mean level of antibodies specific to canine MAGE-B10 was the highest in mice immunized with an rMAGE-B10-HSP110 fusion protein, followed by rMAGE-B10 and a mixture of rMAGE-B10 and rHSP110, respectively. In contrast, rHSP110- or PBS-immunized mice showed low levels of antibodies specific to canine MAGE-B10.

Moreover, the mean level of antibodies specific to canine MAGE-B10 in mice immunized with an rMAGE-B10-HSP110 fusion protein or rMAGE-B10 with adjuvant increased at day 14 and was higher at day 21. Conversely, the mean level of antibodies specific to canine MAGE-B10 in mice immunized with a mixture of rMAGE-B10 and rHSP110 with adjuvant showed an increasing trend at day 7 and continued to rise on days 14 and 21. Comparing the groups at days 14 and 21 post-immunization, the mean level of antibodies specific to canine MAGE-B10 was the highest in mice immunized with a mixture of rMAGE-B10 and rHSP110, followed by rMAGE-B10 and an rMAGE-B10-HSP110 fusion protein (Figure 7). On the other hand, rHSP110 or PBS with adjuvant-immunized mice showed low levels of antibodies specific to canine MAGE-B10.

### 3.5. Cell Activation and Differentiation Response to Protein Candidates In Vitro

**Figure 8:**
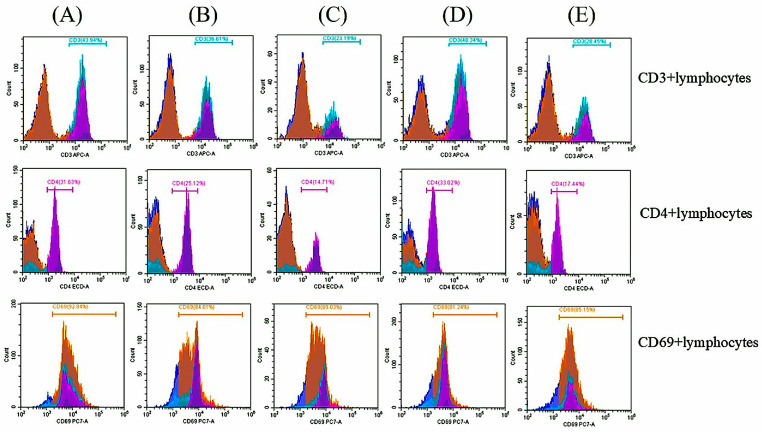
Flow cytometric analysis of T-cell activation markers in immunized mice. Representative histograms illustrate the proportions of CD3+, CD4+, and CD69+ lymphocyte subsets following immunization with the following: (**A**) rMAGE-B10-HSP110 fusion protein, (**B**) rMAGE-B10, (**C**) rHSP110, (**D**) a mixture of rMAGE-B10 and rHSP110, and (**E**) PBS control.

All immunized mice were euthanized 14 days after the second immunization. Single splenocytes were isolated from the immunized mice and assessed for CD3+ and CD4+ activation in each immunization group using FACS analysis. Histogram analyses were performed, and the proportions of target lymphocytes were calculated (Figure 8). The results showed that the mean proportion of CD3+ lymphocytes in total lymphocytes from mice immunized with a mixture of rMAGE-B10 and rHSP110, rMAGE-B10, and an rMAGE-B10-HSP110 fusion protein was significantly higher than that of mice immunized with PBS (*p* = 0.0003, *p* = 0.0104, and *p* = 0.0439, respectively). Similarly, the mean proportion of CD3+ lymphocytes in mice immunized with a mixture of rMAGE-B10 and rHSP110 was significantly higher than in those vaccinated with rHSP110 alone (*p* = 0.0255). However, there was no significant difference in the mean proportion of CD3+ lymphocytes between mice immunized with rHSP110 and those immunized with PBS. Among mice immunized with candidate proteins and adjuvants, no significant difference in the mean proportion of CD3+ lymphocytes were observed between groups.

**Figure 9:**
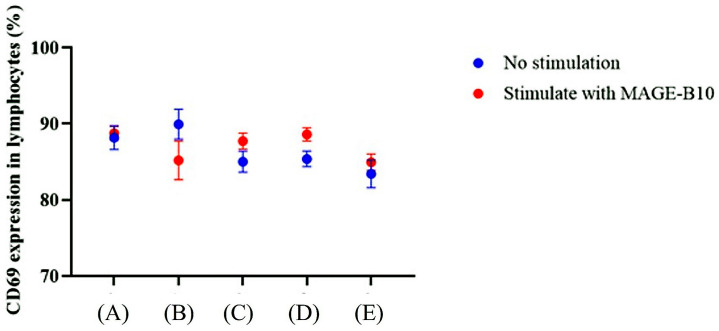
Comparative analysis of CD69+ expression in splenocytes from immunized mice. Splenocytes were isolated from mice vaccinated with various candidates formulated with adjuvant and subjected to ex vivo re-stimulation. The “No Stimulation (blue)” groups were cultured in media alone, while the “Treatment (red)” groups were re-stimulated with purified full-length recombinant canine MAGE-B10 proteins. Groups included the following: (A) rMAGE-B10-HSP110 fusion protein, (B) rMAGE-B10, (C) rHSP110, (D) a mixture of rMAGE-B10 and rHSP110, and (E) PBS control. Data represent the mean level of CD69+ expression.

The mean proportion of CD69+ lymphocytes in total lymphocytes after in vitro stimulation with canine MAGE-B10 proteins was not significantly different compared to unstimulated samples among each group (Figure 9). Among the non-adjuvant groups, the mean proportion of CD69+ lymphocytes in rMAGE-B10-HSP110-fusion-protein-immunized mice was significantly higher than in a mixture of rMAGE-B10- and rHSP110-immunized mice (*p* = 0.0002). In contrast, the mean proportion of CD69+ lymphocytes in a mixture of rMAGE-B10- and rHSP110-immunized mice was significantly lower than in PBS-immunized mice (*p* = 0.0011). Similarly, in the adjuvant group, the mean proportion of CD69+ lymphocytes in rMAGE-B10-immunized mice were significantly higher than in PBS-immunized mice (*p* = 0.035). However, the proportion of CD69+ lymphocytes in total lymphocytes after in vitro re-stimulation with canine MAGE-B10 proteins did not differ significantly among the immunized groups.

### 3.6. IFN-γ-Producing Cells Induced by Protein Candidates In Vitro

**Figure 10:**
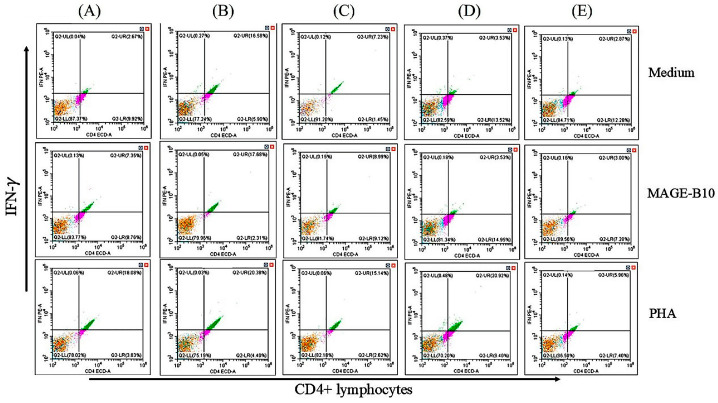
Flow cytometric analysis of intracellular IFN-γ production in CD4+ T lymphocytes. Representative dot plots display the frequency of IFN-γ-secreting CD4+ T cells isolated from mice immunized with the following: (**A**) rMAGE-B10-HSP110 fusion protein, (**B**) rMAGE-B10, (**C**) rHSP110, (**D**) a mixture of rMAGE-B10 and rHSP110, and (**E**) PBS control. Percentages in the [upper right/specified] quadrant represent the IFN-γ-positive population within the gated CD4+ subset.

Single splenocytes were isolated from the immunized mice and re-stimulated with the same purified full-length recombinant canine MAGE-B10 (rMAGE-B10) protein used as the ELISA coating antigen, PHA, and culture medium, serving as the treatment, positive control, and negative control, respectively. Dot plot analysis was performed to evaluate intracellular IFN-γ levels (Figure 10).

The results indicated that the mean proportion of intracellular IFN-γ levels in CD4+ lymphocytes of the total lymphocytes stimulated with purified full-length canine MAGE-B10 (treatment groups) from mice immunized with a mixture of rMAGE-B10 and rHSP110 was significantly higher than in the no-stimulation (medium-only) group (*p* = 0.0488). However, there was no significant difference in the mean intracellular IFN-γ levels in CD4+ lymphocytes between the treatment and control groups in mice immunized with rMAGE-B10, rHSP110, an rMAGE-B10-HSP110 fusion protein, a mixture of rMAGE-B10 and rHSP110, or PBS.

**Figure 11:**
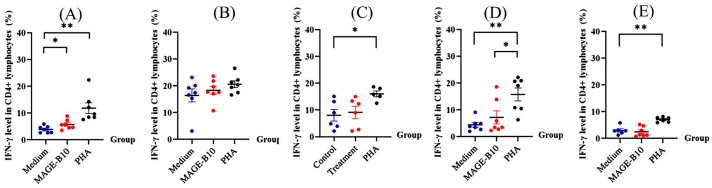
IFN-γ production by CD4^+^ lymphocytes following ex vivo re-stimulation of splenocytes from immunized mice. Splenocytes isolated from mice in each immunization group were re-stimulated ex vivo with culture medium as a negative control, purified full-length canine MAGE-B10 protein for antigen-specific stimulation, or phytohemagglutinin (PHA) as a positive control. The immunization groups were: (**A**) rMAGE-B10-HSP110 fusion protein, (**B**) rMAGE-B10, (**C**) rHSP110, (**D**) a mixture of rMAGE-B10 and rHSP110, and (**E**) PBS. Data are presented as the mean frequency of IFN-γ^+^; cells within the CD4^+^ lymphocyte population. Error bars indicate the standard error of the mean. Asterisks indicate statistically significant differences between the indicated groups (* *p* < 0.05; ** *p* < 0.01).

Among mice immunized with candidate proteins and adjuvant, the mean intracellular IFN-γ levels in CD4+ lymphocytes of total lymphocytes showed no significant difference between the treatment and negative control groups (Figure 11). However, the mean proportion of intracellular IFN-γ levels in CD4+ lymphocytes of total lymphocytes in the PHA-stimulated group (positive control) was higher than in any other group in this study.

## 4. Discussion

Canine mammary tumors are considered invasive, with a high rate of recurrence and metastasis in intact female dogs [6,32,33]. Previous studies have reported that some malignant canine mammary tumor histologic subtypes, including adenosquamous carcinoma, comedocarcinoma, solid carcinoma, anaplastic carcinoma, and carcinosarcoma, are associated with aggressive clinical behavior and unfavorable outcomes. In particular, anaplastic carcinoma and carcinosarcoma have been reported to show very high metastatic rates, although prognosis should be interpreted together with other clinicopathological parameters rather than histologic subtype alone [33,34]. Innovative treatments focusing on targeted molecular therapy with fewer adverse effects as alternatives to surgery are preferred. Tumor vaccines have been developed over the past years [35,36]. The MAGE gene family has been identified as an attractive target for cancer vaccines, aiming to stimulate immune responses and target MAGE antigen-expressing tumor cells [37,38,39]. Our preliminary study indicated that MAGE-B10 exhibits high expression in these two tumor types and is an attractive immunotherapeutic target gene [25]. The present study prepared a fusion protein prototype against canine MAGE-B10-expressing tumors using MAGE-B10 and HSP110 and investigated their efficacy in inducing innate and adaptive immune responses specific to canine MAGE-B10 protein in vitro. A limitation of the present study is that the target disease is canine mammary tumor, whereas the functional immunogenicity data were generated in mice. Thus, the findings should be interpreted as a preliminary proof-of-concept for immunogenicity rather than as direct evidence of vaccine efficacy in dogs. In particular, interspecies differences in major histocompatibility complex (MHC) molecules, including differences between dog leukocyte antigen (DLA) and murine H-2, may affect antigen processing, epitope presentation, and T-cell activation. In addition, the degree of homology between canine HSP110 and murine HSP110 may reduce the apparent immunogenicity of the HSP110 component in the mouse model. Therefore, further studies in canine systems are required to confirm the translational relevance of this vaccine candidate.

Due to their economical and fast-growing properties, *E. coli* is one of the most frequently used cell systems for producing recombinant proteins. However, recombinant proteins are sometimes unsuccessful in bacterial systems, and some may not be properly folded, post-translationally modified, or translocated. This can reduce solubility and activity, resulting in inclusion body formation and hindering bacterial growth [40,41]. Previous studies have indicated that heat shock proteins (HSPs), a major group of molecular chaperones involved in stress resistance and proper protein folding, are frequently used as expression partners or co-expressed with recombinant proteins [42]. The targeted canine MAGE-B10 fragment (amino acids 55–232; NCBI database accession no. NP_001003116), used in the present study for fusion protein production, was not successfully recovered as a stable standalone recombinant protein in the pGEX-4T-1/BL21 E. coli expression system. This likely reflected poor solubility, improper folding, instability, or rapid degradation of the expressed product rather than absence of coding potential. However, after linking this target canine MAGE-B10 fragment with the highly conserved domain of HSP110, the rMAGE-B10-HSP110 fusion protein was readily expressed and recovered using pGEX-4T-1. A possible explanation is that HSP110, as a heat shock protein with chaperone-related properties, improved the folding, solubility, or stability of the fusion product and thereby enhanced recombinant protein recoverability in the bacterial expression system. In this study, the MAGE-B10 sequence used in the rMAGE-B10-HSP110 fusion construct corresponded to a highly conserved 539 bp region, whereas the comparative rMAGE-B10 protein was generated from the full-length 1134 bp cDNA. This difference was due to technical limitations of the bacterial expression system. The selected conserved MAGE-B10 fragment could not be successfully expressed alone in the pGEX-4T-1/BL21 *E. coli* system, whereas fusion with HSP110 enabled successful recombinant protein production. Therefore, full-length rMAGE-B10 was used as the comparator because it was the feasible standalone recombinant MAGE-B10 protein in our system. We acknowledge that the unequal MAGE-B10 sequence lengths may influence the breadth of antigenic epitopes presented and thus represent a limitation of the present study. However, because antibody detection and splenocyte re-stimulation were performed using recombinant canine MAGE-B10 antigen, the observed responses indicate that the conserved MAGE-B10 region included in the fusion protein retained immunologically relevant epitopes. Future studies should directly compare conserved-region and full-length MAGE-B10-HSP110 fusion constructs if full-length fusion expression can be achieved. However, after linking the target canine MAGE-B10 gene with the highly conserved domain of the HSP110 gene, the rMAGE-B10-HSP110 fusion protein was readily expressed using pGEX-4T-1. In contrast, in the present study, full-length canine MAGE-B10 and the targeted highly conserved domain of the HSP110 fragment (amino acids 492–788; NCBI database accession no. XP_013962905) used for comparative proteins had no expression problems when using pGEX-4T-1 and the *E. coli* system. This may be associated with the HSP properties in co-expression, as many studies report the positive effects of chaperone gene co-expression regarding solubility, yield, secretion ability, and specific activity [43,44,45,46].

The innate and adaptive immune systems constantly interact to generate an efficient immune response. In the early phase of immune responses, the innate immune system binds and activates through pattern recognition receptors (PRRs) expressed on antigen-presenting cells (APCs), and the adaptive immune response follows. Prior studies also highlighted the prominent feature of the MAGE-A3 protein vaccine, which could induce strong antibody responses against tumor antigens [39]. The antibody response study reflected the innate link to adaptive immune responses that eventually activate B cell progenitors in the production of antibodies specific to the immunogen. The present study’s results suggest that the rMAGE-B10-HSP110 fusion protein and rMAGE-B10 were able to induce naïve B cells to proliferate into plasma cells or memory B cells and produce canine MAGE-B10-specific antibodies in mouse sera. These events also support the observation that the selective fragment of MAGE-B10 used for fusion protein construction was acceptable. However, the identity of the protective epitope(s) within the canine MAGE-B protein should be further analyzed.

The CD3+ antigen is a surface structure associated with the T-cell receptor (TCR), forming a complex involved in T-cell-mediated immune responses, such as antigen recognition, signal transduction, and activation of immunocompetent T lymphocytes. For this reason, CD3+ is present at all stages of T-cell development and is a highly effective T-cell marker [47,48]. The present study highlighted that canine MAGE-B10 has immunogenic properties in mice, as the proportion of CD3+ lymphocytes among total lymphocytes was higher in mice that received the rMAGE-B10-HSP110 fusion protein, rMAGE-B10 alone, or a mixture of rMAGE-B10 and rHSP110 than in those that did not. In contrast, canine HSP110 had fewer properties as an immunogen that stimulates the pathway leading to CD3+ lymphocyte activation and proliferation compared with the canine MAGE-B10 immunogen. This could be explained in terms of the homology of peptide sequences between species. The fragment of canine HSP110 in the present study is 89.56% similar to that of mice (National Center for Biotechnology Information, Bethesda, MD, USA). This may mimic the self-antigen of mice, which the immune system may eliminate, and to which T lymphocytes may not respond. In addition, CD4+ is a glycoprotein that serves as a co-receptor for the T-cell receptor (TCR). CD4+ lymphocytes, or T helper cells, play an important role in the adaptive immune system [49]. This study revealed that an rMAGE-B10-HSP110 fusion protein with and without adjuvant could activate transduction between innate and adaptive immunity, resulting in CD4+ lymphocyte proliferation.

Immunotherapy utilizing T cells that attack tumors is a promising strategy for treatment, but immune-suppressive T cells, such as regulatory T cells (Tregs), and immune checkpoint molecules, including programmed death-1 (PD-1), can suppress the intensity of T-cell immune reactions and impair tumor clearance. CD69+ is a membrane-bound, type II C-lectin receptor well known as a classical early marker of lymphocyte activation and mainly used as a marker of activated lymphocytes (cytotoxic T cells, memory T cells, helper T cells, and Tregs) and NK cells [50]. CD69+ has a function in immune suppression in the tumor microenvironment by regulating Treg cell activation, differentiation, tissue retention, and dysfunction [51,52]. According to the results, the MAGE-B10-HSP110 fusion protein effectively induced antigen-specific cellular immune responses via CD69+ lymphocytes. A limitation of the present flow cytometric analysis is that CD3+, CD4+, and CD69+ populations were evaluated as separate parameters, while double-positive activated T-cell subsets such as CD3+/CD69+ and CD4+/CD69+ were not separately analyzed. Future studies should include these subset analyses to better define the activated immune cell populations induced by the vaccine candidates. Interestingly, the proportion of CD69+ lymphocytes in total lymphocytes in mice that received rHSP110 with and without MAGE-B10 protein tended to be lower than in other groups of mice. The regulation of this CD69+ expression lymphocyte related to HSP110 should be further studied. However, the differentiation of T-cell progenitors to activated T cells after splenocyte restimulation with MAGE-B10 in vitro did not result in a significant change in the proportion of CD69+ expression T cells measured. The differentiation of T cells needs key cytokines and transcription factors, which are produced by cells located outside the spleen [53,54]. Due to the limitation of these factors in in vitro cell environments, these arguments should be investigated further in in vivo mouse models.

IFN-γ is a dimerized soluble cytokine critical to the adaptive immune response to intracellular immunogens and tumors [55]. The level of intracellular IFN-γ produced by splenic lymphocytes, including helper T cells, cytotoxic T cells, and NK cells, was evaluated. This study evaluated the IFN-γ mainly produced by helper T cells, and the results revealed that rMAGE-B10-HSP110 fusion proteins could induce cytokine production after being re-stimulated with the rMAGE-B10 immunogen in vitro, reflecting the transduction between innate and adaptive immune responses. A limitation of the present flow cytometric analysis is that intracellular IFN-γ responses were interpreted primarily from the proportion of IFN-γ-positive cells, whereas mean fluorescence intensity (MFI) values were not separately extracted and reported. Future studies should include MFI-based analysis to provide additional quantitative information on cytokine expression levels at the single-cell level. Prior studies indicated that MAGE-B-specific CD8+ T cells secrete IFN-γ, as tumor cell killing is mediated through CD8+ T cell function, and CD4+ T cells secreting IFN-γ may play an important role in tumor rejection [56,57]. Similarly, other murine MAGE-B vaccine studies have found that MAGE-B-vaccine-immunized mice could induce these IFN-γ responses to the MAGE-B antigen [58,59]. However, in the present study, statistically significant antigen-specific increases in intracellular IFN-γ were not consistently observed across all immunized groups and were mainly evident in the mixture group under certain conditions. Therefore, the IFN-γ findings should be interpreted cautiously and considered a limitation of this preliminary study. Further investigation using expanded sample sizes and additional functional assays will be needed to confirm the consistency and biological relevance of antigen-specific cellular responses induced by the vaccine candidates.

HSP110 has demonstrated immunoadjuvant properties that could induce danger signals and pro-inflammatory cytokines in mouse mammary carcinoma through interactions with antigen-presenting cells [24,60]. According to the results, the fragment of HSP110 used in fusion protein prototype production uncovered the ability to alert danger signals linked to the adaptive immune system, not differing from the montanide adjuvant-added fusion protein. However, the present study was a preliminary study of the fusion protein prototype against MAGE-B10-expressing tumors in vitro. Further studies into their immune responses require more investigation in vivo.

## 5. Conclusions

This study discovered a new fusion protein prototype for MAGE-B10-expressing CMTs by linking HSP110 with MAGE-B10, designated as an rMAGE-B10-HSP110 fusion protein. Additionally, the potency of this protein was investigated. The results highlighted that an rMAGE-B10-HSP110 fusion protein could stimulate innate immunity and induce a transition to adaptive immunity in vitro. A high level of antibody titer specific to canine MAGE-B10 was observed in mice immunized with an rMAGE-B10-HSP110 fusion protein. Similarly, intracellular IFN-γ in CD4+ lymphocytes were observed in the splenocytes of an rMAGE-B10-HSP110-fusion-protein-immunized mouse after stimulation with canine MAGE-B10. Moreover, the proportions of CD3+, CD4+, and CD69+ lymphocytes in the total lymphocytes of immunized mice were illustrated, and they reflected how the immune system responds to the candidate recombinant proteins. Further studies in canine-relevant systems are needed to confirm its translational applicability, antitumor efficacy, and potential for preventing tumor recurrence after surgery.

## Figures and Tables

**Table 1:** The canine MAGE-B10 primer pairs.

Primer Name	Sequences	Product Size (bp)
cMAGE-B10-F	5′-TATAGCGTTTCCCAGGGTCCTC-3′	539
cMAGE-B10-R	5′-TCCATCCCCGCATATAACCCAA-3′	
PGEX-mage-F	5′-ATGAATTCATGCCGCGGGGTCAGAAGAGTAAGC-3′	1134
PGEX-mage-R	5′-ATGCGGCCGCTAGACTTTATTAGGGGTGGGAGGAATT-3′	
PGEX-5′	5′-GGGCTGGCAAGCCACGTTTGGTG-3′	1306
PGEX-3′	5′-CCGGGAGCTGCATGTGTCAGAGG-3′	

**Table 2:** The immunizations.

Immunogens	Description	Concentration (pmol/mL)		Vaccine Formulation (mL)		
		Initial	Final	Protein	Adjuvant	Total
1. rMAGE-B10-HSP110 fusion protein	rMAGE-B10-HSP110 fusion protein with adjuvant	400	200	0.1	0.1	0.2
2. rMAGE-B10	rMAGE-B10 with adjuvant	400	200	0.1	0.1	0.2
3. rHSP110	rHSP110 with adjuvant	400	200	0.1	0.1	0.2
4. A mixture of rMAGE-B10 and rHSP110	rMAGE-B10 with adjuvant	800	200	0.05	0.05	0.1
	rHSP110 with adjuvant	800	200	0.05	0.05	0.1
5. PBS	PBS with adjuvant	-	-	0.1	0.1	0.2

## Data Availability

The dataset is available from the authors upon request. The raw data supporting the conclusions of this article will be made available by the authors upon request.

## Supplementary Materials

The following supporting information can be downloaded at: https://www.mdpi.com/article/10.3390/ani16091374/s1, Figure S1: SDS-PAGE analysis of rMAGE-B10 protein expression. Total cell lysates from *E. coli* BL21 were analyzed under varying IPTG concentrations and induction durations. The arrows indicate the target rMAGE-B10 protein at the expected molecular weight of approximately 68 kDa. For both panels: Lane M: protein molecular weight marker (kDa); Lane 1: non-induced *E. coli* BL21 control; Lanes 2–4: lysates induced with 0.5, 1.0, and 3.0 mM IPTG for 6 h, respectively; Lanes 5–7: lysates induced with 0.5, 1.0, and 3.0 mM IPTG for 24 h, respectively. Figure S2: Western blot analysis of rMAGE-B10 protein expression. Total cell lysates from *E. coli* BL21 were analyzed under varying IPTG concentrations and induction durations. The arrows indicate the target rMAGE-B10 protein at the expected molecular weight of approximately 68 kDa. For both panels: Lane M: protein molecular weight marker (kDa); Lane 1: non-induced *E. coli* BL21 control; Lanes 2–4: lysates induced with 0.5, 1.0, and 3.0 mM IPTG for 6 h, respectively; Lanes 5–7: lysates induced with 0.5, 1.0, and 3.0 mM IPTG for 24 h, respectively.

## Abbreviations

The following abbreviations are used in this manuscript:

A mixture of rMAGE-B10 and rHSP110	A mixture of recombinant MAGE-B10 and recombinant HSP110 protein
APC	Allophycocyanin
CMTs	Canine mammary tumors
FACS	Fluorescence-activated cell sorting
GST	Glutathione S-transferase
HSP110	Heat shock protein 110
IPTG	Isopropyl β-D-1-thiogalactopyranoside
MAGE	Melanoma-associated antigen
PBS	Phosphate-buffered saline
PHA	Phytohaemagglutinin
rHSP110	A recombinant HSP110 protein
rMAGE-B10	A recombinant MAGE-B10 protein
rMAGE-B10-HSP110 fusion protein	A fusion recombinant MAGE-B10 and HSP110 protein
TMB	Tetramethylbenzidine
