# Supplementary material for: A Novel Recombinant MAGE-B10-HSP110 Fusion Protein Enhances Innate and Adaptive Immune Responses in Mice: A Potential Vaccine Candidate for Canine Mammary Tumors
Source: Animals (Basel). 2026 Apr 29;16(9):1374. doi: 10.3390/ani16091374 (PMC13163047; doi:10.3390/ani16091374)
Supplement: Supplementary file 1 [file animals-16-01374-s001.zip › Supplementary Figure.pdf]

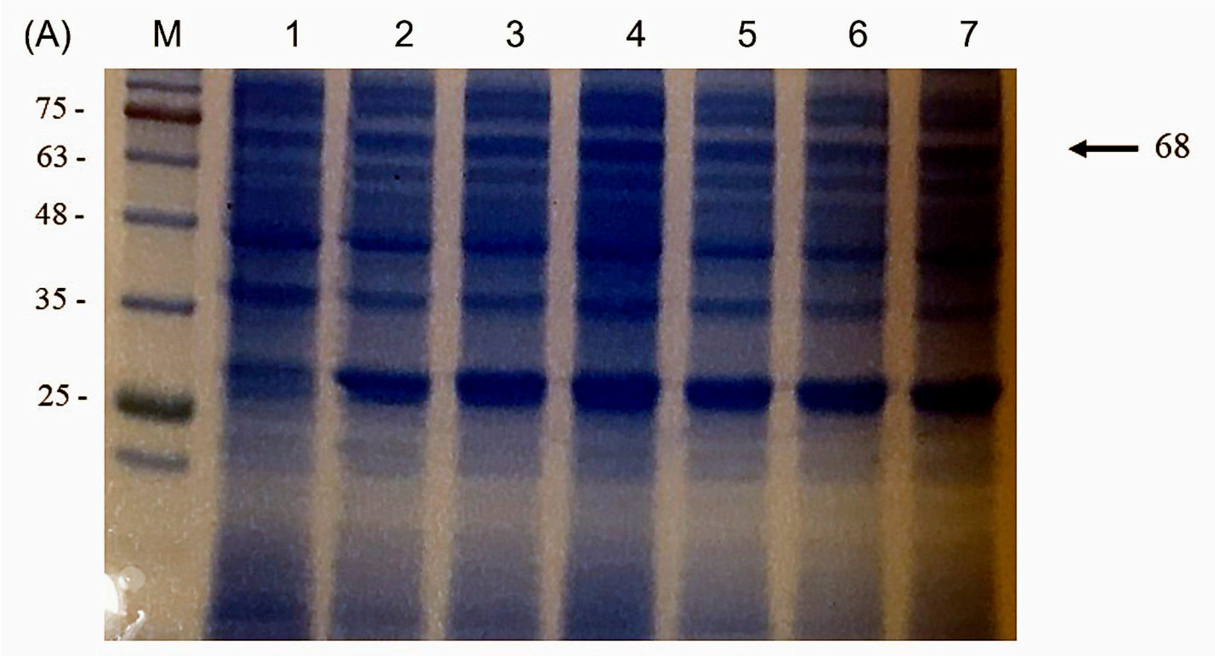

Supplementary Figure S1. SDS-PAGE analysis of rMAGE-B10 protein expression. Total cell lysates from *E. coli* BL21 were analyzed under varying IPTG concentrations and induction durations. The arrows indicate the target rMAGE-B10 protein at the expected molecular weight of approximately 68 kDa. For both panels: Lane M: protein molecular weight marker (kDa); Lane 1: non-induced *E. coli* BL21 control; Lanes 2–4: lysates induced with 0.5, 1.0, and 3.0 mM IPTG for 6 h, respectively; Lanes 5–7: lysates induced with 0.5, 1.0, and 3.0 mM IPTG for 24 h, respectively.

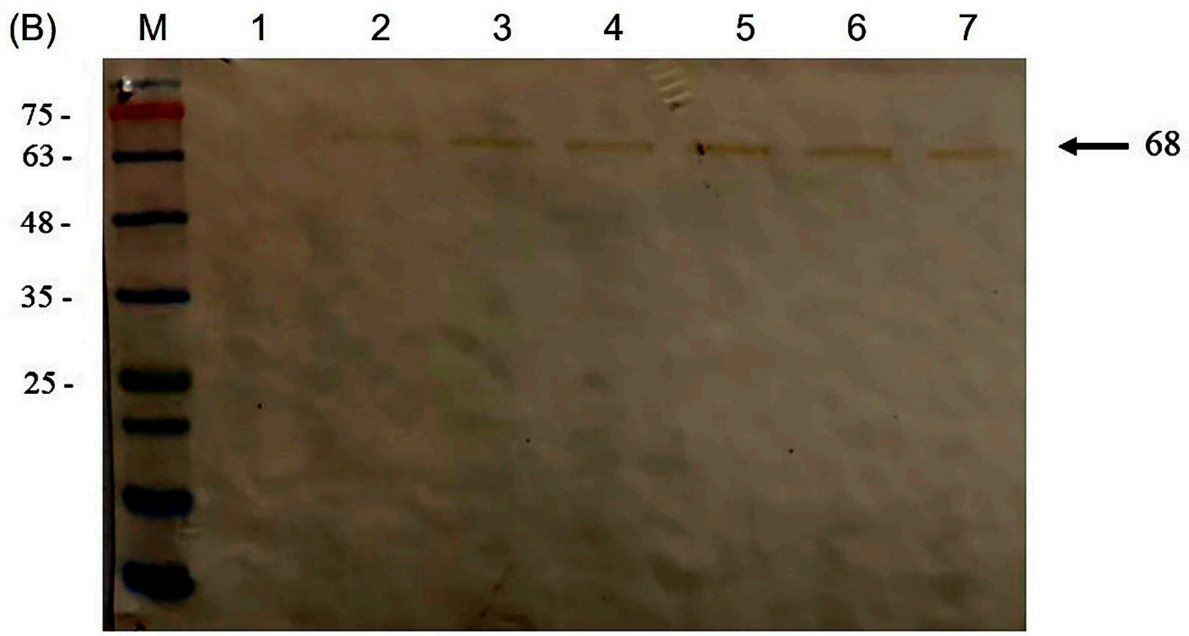

Supplementary Figure S2. Western blot analysis of rMAGE-B10 protein expression. Total cell lysates from *E. coli* BL21 were analyzed under varying IPTG concentrations and induction durations. The arrows indicate the target rMAGE-B10 protein at the expected molecular weight of approximately 68 kDa. For both panels: Lane M: protein molecular weight marker (kDa); Lane 1: non-induced *E. coli* BL21 control; Lanes 2–4: lysates induced with 0.5, 1.0, and 3.0 mM IPTG for 6 h, respectively; Lanes 5–7: lysates induced with 0.5, 1.0, and 3.0 mM IPTG for 24 h, respectively.
